# An update on the ophthalmic features in hereditary haemorrhagic telangiectasia (Rendu-Osler-Weber syndrome)

**DOI:** 10.1007/s10792-021-02197-y

**Published:** 2022-01-16

**Authors:** Solmaz Abdolrahimzadeh, Martina Formisano, Carla Marani, Siavash Rahimi

**Affiliations:** 1 Ophthalmology Unit, Neurosciences, Mental Health and Sense Organs (NESMOS) Department, University of Rome Sapienza, Rome, Italy; 2 Faculty of Medicine and Psychology, St. Andrea Hospital, Via di Grottarossa 1035/1039, 00189 Rome, Italy; 3 Department of Sense Organs, Ophthalmology Unit, University of Rome Sapienza, Azienda Policlinico Umberto I, viale del Policlinico 155, 00161 Rome, Italy; 4 San Carlo Hospital, Via Aurelia 275, 00165 Rome, Italy; 5 Istituto Dermopatico dell’Immacolata (IDI-IRCCS) Department of Histopathology, Via Monti di Creta 104, 00167 Rome, Italy

**Keywords:** Hereditary haemorrhagic telangiectasia, Ophthalmic, Rendu-Osler-Weber syndrome, Wide-field fluorescein angiography, Multimodal imaging, Near infrared reflectance, Spectral domain optical coherence tomography

## Abstract

Hereditary haemorrhagic telangiectasia (HHT) or Osler-Rendu-Weber syndrome is a rare autosomal dominant disease, characterised by systemic angiodysplasia. Dysfunction of the signalling pathway of β transforming growth factor is the main cause of HHT principally owing to mutations of the genes encoding for endoglin (ENG) and activin A receptor type II-like 1 (ACVRL1). Clinical manifestations can range from mucocutaneous telangiectasia to organ arterio-venous malformations and recurrent epistaxis. The early clinical manifestations may sometimes be subtle, and diagnosis may be delayed. The main ophthalmic manifestations historically reported in HHT are haemorrhagic epiphora, and conjunctival telangiectasia present in 45–65% of cases, however, imaging with wide-field fluorescein angiography has recently shown peripheral retinal telangiectasia in 83% of patients. Optimal management of HHT requires both understanding of the clinical presentations and detection of early signs of disease. Advances in imaging methods in ophthalmology such as wide-field fluorescein angiography, spectral domain optical coherence tomography, and near infrared reflectance promise further insight into the ophthalmic signs of HHT towards improved diagnosis and early management of possible severe complications.

## Introduction

**Fig. 1:**
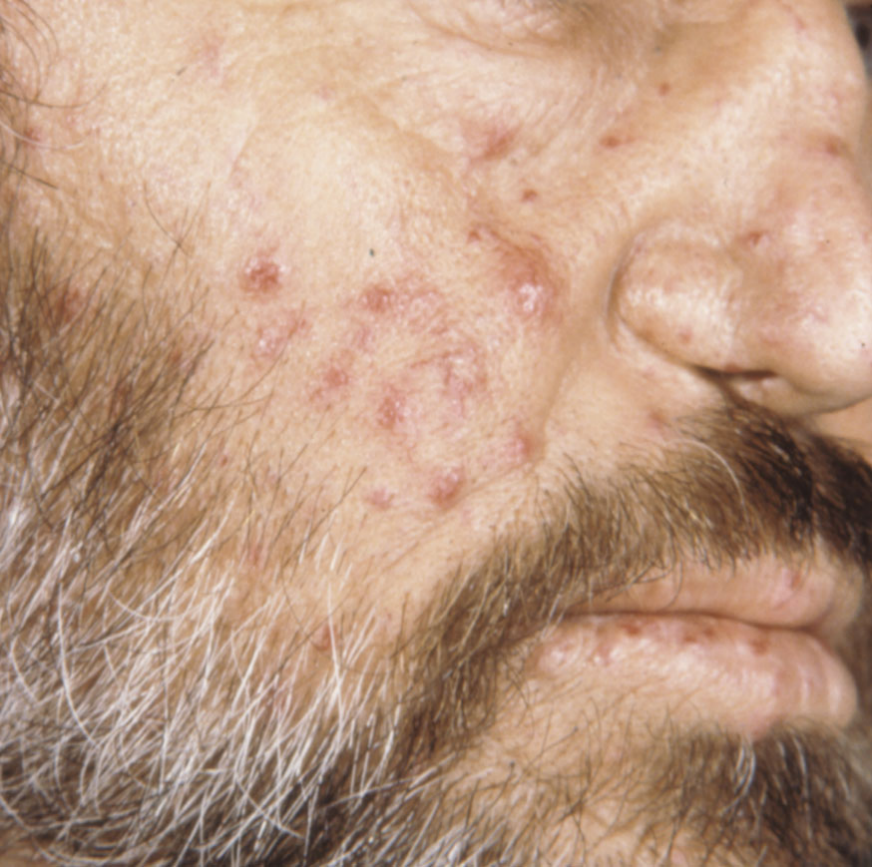
Telangiectasia of the facial skin and lips in a patient with hereditary haemorrhagic telangiectasia. Reproduced with permission from: Recupero SM, Abdolrahimzadeh S, Lepore G, et al. L’apparato oculare nelle sindromi neurocutanee. Rome: Verduci Editore 2004 [63]

Hereditary haemorrhagic telangiectasia (HHT) or Rendu-Osler-Weber syndrome is a rare systemic fibrovascular dysplasia with an incidence in the general population of 1–2/5000–8000 [1]. The disease affects the elastic and muscle layers of the vessel walls with a consequent increased susceptibility to ruptures and bleeding episodes [2]. HHT is characterised by multiple arterio-venous malformations (AVMs) where arteries and veins are connected in the absence of capillaries. Small AVMs are called telangiectasia, and large forms can be greater than a few millimetres up to several centimetres in diameter [3]. Transmission is autosomal dominant with incomplete expression in some cases, and sporadic mutations are involved in 20% of cases [4]. Mutations of endoglin (ENG) and activin A receptor type II-like 1 (ACVRL1) account for about 85% of cases in HHT type I and II, respectively. A third locus has been reported for HHT type III, and HHT type IV involves mutations of the SMAD4 gene that can manifest in the rare form of juvenile polyposis and (JHHT) [5–7]. Manifestations range from multiple mucocutaneous telangiectasia involving the lips, oral cavity (tongue, palate), nasopharynx, conjunctiva, ears, skin, fingers (fingertips, nail folds), arms, and trunk to AVMs in major organs such as the lung, brain, gastrointestinal tract, liver, and spinal cord [8]. (Fig. 1) Quality of life is compromised by recurrent bleeding, and morbidity is due to haemorrhage, stroke, aneurysms, and cerebral abscesses [9–11]. Initial diagnosis is frequently made based on recurrent epistaxis, oral lesions discovered during routine dental examination, and ocular signs [12, 13]. Ophthalmic manifestations have historically been described in 45–65% of patients with conjunctiva telangiectasia and haemorrhagic epihora, but recently advanced imaging modalities have shown peripheral retinal telangiectasia in 83% of cases [14–17].

HHT shows age-related penetrance and clinical manifestations develop over time. Careful multidisciplinary examination may reveal telangiectasia early in life. Awareness of HHT and early diagnosis is crucial to avoid the pitfalls of potentially severe complications in routine medical practice. The present review is focused on the ophthalmic manifestations of HHT in view of novel imaging methods.

## History

Recurrent epistaxis, telangiectasia on the torso and the face, and lesions on the soft palate and lips were first identified by the French physician Henri Jules Louise Marie Rendu (24 July, 1844 -16 April, 1902) in 1896 [18]. Sir William Osler (July 12, 1846-December 29, 1919) a Canadian physician and bibliophile, described disease inheritance, punctiform angiomata on facial skin and mucosa of the lips, cheeks, tongue, and nose with recurring epistaxis and visceral involvement of the stomach in 1901 [19]. Frederick Parkes Weber (8 May, 1863–2 June, 1962), an English dermatologist, reported a case series demonstrating the association between bleeding, mucocutaneous lesions, AVMs, and conjunctiva telangiectasia in 1907 [20]. Retinal angiomata and retinal bleeding were described in “Osler’s disease” by Francois and Landau [21, 22].

## Epidemiology

There is wide geographic variability however, the highest prevalence is among the Afro-Caribbean population of the island of Curaçao and Bonaire (1 case in 1331 inhabitants) from which derive the Curacao diagnostic criteria [23, 24]. HHT type 1 is more frequent in North America and in Northern Europe. HHT type 2 more prevalent in Mediterranean countries such as Italy, France, and Spain [25–30].

## Pathogenesis

Rare diseases are currently studied and classified using molecular genetics, and HHT is not an exception to this trend. Our understanding of the pathogenesis of rare diseases such as HHT is constantly increasing with molecular diagnostic methods that enable correct and precise classification, nevertheless, the knowledge of the clinical features of HHT is essential for the practising clinician.

The genes implicated in the disease are critical for blood vessel development and response to TGF-β signalling [3]. Mutations causing HHT type I and II are commonly of Endoglin (ENG) on chromosome 9q33-34, and Activin A Receptor Like Type 1 (ACVRL-1) receptor on chromosome 12q13, respectively. Both genes code a membrane glycoprotein that is expressed in endothelial tissue cells and make up the surface receptor for TGF-β that mediates vascular remodelling by affecting extracellular matrix production. ENG, ACVRL – 1, and TGF-β function are essential for angiogenesis [6]. SMAD4 gene mutation is not included in the currently used diagnostic criteria for HHT but is tested in cases of strong clinical suspicion of HHT that do not fulfil diagnostic criteria, and when testing family members of HHT patients [31]. This mutation is linked to a rare syndrome of combined juvenile polyposis and hereditary haemorrhagic telangiectasia JHHT [1]. A new locus for HHT (HHT type III) has been mapped to chromosome 5 and a fourth locus to chromosome 7 [7, 32].

Gòmez-Acebo et al. evaluated 206 Spanish patients with HHT and found that patients with ocular involvement had a more frequent mutation for the ENG gene rather than the ACVRL1/ALK1 gene. In contrast, Letteboer et al. reported conjunctival involvement in 13.4% of patients with HHTI and 14.6% in patients with HHTII [33].

## Clinical manifestations

**Fig. 2:**
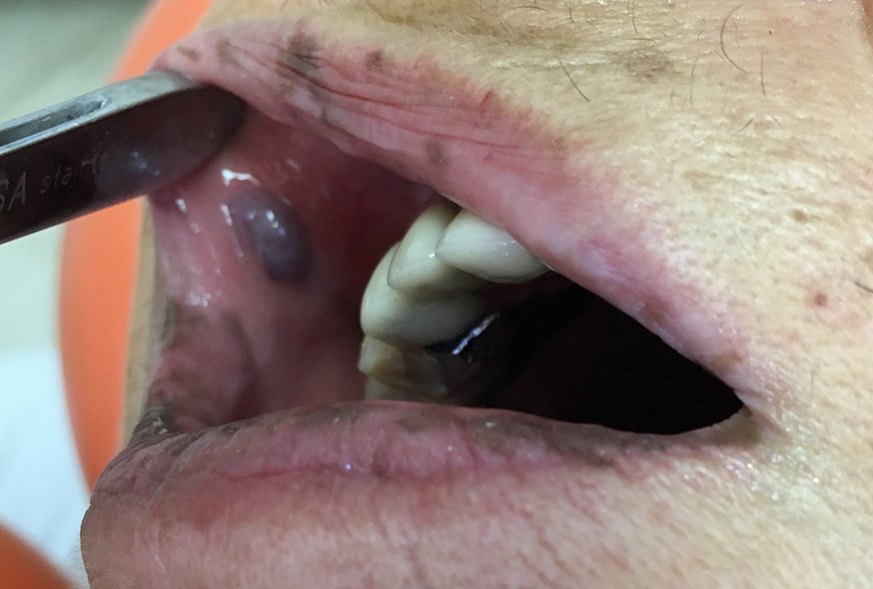
Oral manifestations of hereditary haemorrhagic telangiectasia. (Courtesy of Dr. Gianmarco Bellardini)

The vascular alterations of HHT are present in numerous organ systems of the body and are characterised by multiple capillary and venule dilatations that are fragile and can haemorrhage owing to thin vessel walls. The otorhinolaryngology specialist is often the first clinician called to make a diagnosis of HHT as recurrent epistaxis has been described as the main and first manifestation, present in 90% of patients as early as 10 years of age [2]. Mucocutaneous telengiectasia occur in about 90% of cases of HHT [34]. Histologically, they appear as a superficial set of dilated blood vessels lacking perivascular elastic fibres and smooth muscle [35]. Multiple telangiectasia may be pin-point sized or larger and spiderlike in elderly patients. Spider telangiectasia are often found in the mucous membranes of the nose, oral cavity, skin of the face and neck and increase with age [36]. Buckmiller et al. [37] reported that AVM lesions may appear with an overlying vascular blush hue in the skin akin to an early naevus flammeus [38].When the mucosa is involved, it is usually thickened and vascular. The more advanced lesions may present enlarged vessels in the skin and underneath and non-compressible, thickened tissue with pulsation. AVMs can progress with consequent ulcerations and bleeding [37]. HHT may present with haemorrhagic vesicles, nodules, papules, or ulcers of varying size involving the gingiva, [35] the oral mucosa in the region of the tongue, hard palate, and vermilion of the lip [13]. (Fig. 2) Patients may experience intense bleeding from tooth brushing due to poor oral health and gingival disease [39]. Pathological life-threatening oral bleeds are rare but the most common are related to AVMs of the jaws or facial trauma in haemophiliac patients [40].

Formal diagnosis of HHT is made according to the Curaçao criteria: telangiectasia on the face, hand and oral cavity, recurrent epistaxis, arterio-venous malformations with visceral involvement, and familial history [23, 24]. Diagnosis is suspected when two criteria are met, whereas it is considered definite with at least three of four criteria. Following confirmed diagnosis of HHT complementary imaging tests such as computed tomography, ultrasound, and magnetic resonance imaging are advised to detect eventual involvement of organs [41–43]. Table 1 is a summary of the findings and symptoms in organ involvement. However, approximately 20% of patients are not aware of family history of disease, and only have mild symptoms [44]. Li et al. and Pierucci et al. reported an average diagnostic delay of 26 years [45–47]. The reasons behind this delay are most probably due to the rare presentation of HHT, because symptoms are variable and age dependent, and their onset is usually in the fourth (90%) or sixth decade (97%) of life [1].

**Table 1:** Organ involvement in hereditary haemorrhagic telangiectasia. (AVM: arterio-venous malformations)

Organ	Findings	Symptoms	References
Brain	Cerebral AVM (5–15%)	headache, migraine, brain abscess, seizures, paraparesis, ischaemia, stroke, transient ischaemic attacks, intracerebral and subarachnoid haemorrhage	Sabba [7]
			Letteboer [26]
			Shovlin [42]
			Sadick [34]
			Begbie [43]
	Spinal AVM	subarachnoid haemorrhage, seizures, paraparesis	
Lungs	Pulmonary AVMs (15–30%)	right-to-left shunt with possible secondary chronic hypoxemia and paradoxical embolism	Sadick [34] Guttmacher [11]
Digestive system	Gastrointestinal AVM (Gastrointestinal bleeding 20–40%)	gastrointestinal bleeding with possible iron deficiency anaemia or acute gastrointestinal haemorrhage	Begbie [44]
Liver	Liver AVM (Liver telangiectasia 8–31%)	portal hypertension, high output heart failure, biliary disease, shunt between hepatic arteria and portal vein and consequent pseudocirrhosis	Daina [41]

Increased knowledge of the disease and a multidisciplinary approach can potentially accelerate diagnosis and consequently improve the quality of life of patients. Furthermore, early diagnosis is important as organ involvement, later in life, can be sudden, acute, severe, and life-threatening.

### Ophthalmic manifestations

Ocular manifestations have historically been described in 35 to 65% of patients with HHT [14–17]. There have been some reports of ocular symptoms leading to the primary diagnosis of HHT through ophthalmologic examination [44, 45]. Recent advances in ophthalmology and the advent of multimodal imaging have improved evaluation of ophthalmic signs as retinal telangiectasias were recently reported in 83% of patients with HHT [48].

#### Conjunctiva

**Fig. 3:**
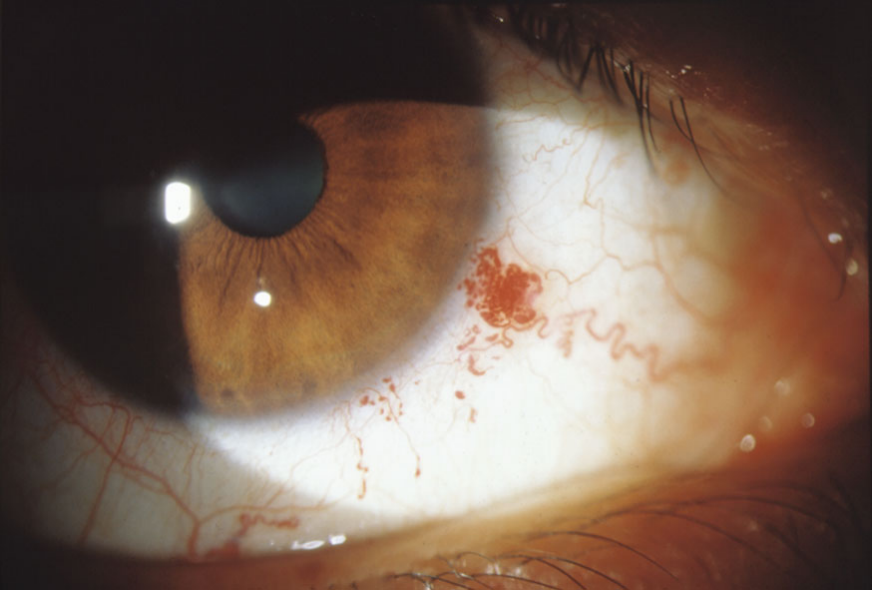
Conjunctival telangiectasia in a patient with hereditary haemorrhagic telangiectasia. Reproduced with permission from: Recupero SM, Abdolrahimzadeh S, Lepore G, et al. L’apparato oculare nelle sindromi neurocutanee. Rome: Verduci Editore 2004:71 [63]

The frightening occurrence of bloody tears can cause considerable alarm in patients who have not yet been diagnosed. Careful slit lamp examination can reveal the cause as conjunctival telangiectasia and lead to comprehensive multidisciplinary evaluation of patients (Fig. 3). Haemorrhagic epiphora and conjunctival telangiectasia have a prevalence of 35–42%. [14–16].

Geisthoff et al. found conjunctival telangiectases in 28 of 74 patients (38%) (47 of 148 eyes). The mean age of patients was 60 years. These authors calculated the precise number of conjunctival telangiectasia and found a maximum number of 15 in each eye with a median of 2. They did not find any correlation between age and number of conjunctival telangiectasia [15]. Gomez-Acebo et al. studied the palpebral and conjunctival location of telangiectasia. They reported upper and lower, upper, and lower eyelid localization in 30, 42, and 32 eyes, respectively. Congiuntival location was prevalently in the tarsal area in 150 eyes; and bulbar, of the border, and caruncle in 2, 7, and 1 eye, respectively [49]. Thus, the frequency of conjunctival telangiectasia can be underestimated unless thorough congiuntival examination is carried out with eyelid eversion.

Differential diagnosis of HHT related bleeding includes haematological disorders such as haemophilia, conjunctivitis, hysterical psychosis, vascular tumours, granulomas, post-surgical ocular conditions or manipulation, naslolacrimal duct pathologies with regurgitant flow to the conjunctiva, and extreme physical exertion [50]. Of interest is the report by Knox who reported a patient with injection of the medial bulbar conjunctiva, where initial diagnosis was episclerits [44]. The authors described an absence of response to topical prednisolone and because the vessels were mobile over the underlying episclera, more detailed history and examination led to diagnosis of conjunctival telangiectasia and HHT.

Contrasting results were reported for correlations between conjunctival and visceral manifestaions of HHT. Geisthoff et al. used the Curaçao criteria to evaluate possible correlations but found that the percentage of patients with visceral involvement was similar for patients with conjunctival telangiectasia (39%) and those without (41%) [15]. On the other hand, Gòmez-Acebo et al. found that conjunctival telangiectasia in HHT were associated with lung involvement and suggested that conjunctival telangiectasias could be a non-invasive marker of HHT manifestations of the lung. These authors also found that oral haemorraghes and grade of telangiectasia of the nose and intensity of epistaxis are also associated with conjunctival telangiectasia [49].

There are no reports of specific treatment modalities for conjunctival telangiectasia, or conjunctival post-haemorrhagic granulomatous lesions as haemorrhage is usually not severe [8]. The rare case reports of cautery for recurrent haemorrhages were reported by Garner et al. and Pandolfi et al. in 1956, and 1978, respectively [51, 52]. Brant et al. in 1989 also reported one case of conjunctival telangiectasia on the upper eyelid that necessitated thermal cautery because of multiple episodes of bleeding [14].

Although bleeding owing to conjunctival telangiectasia is not severe, once HHT is confirmed by molecular diagnosis, patients with history of gastrointestinal bleeding, frequent epistaxis, or possibly frequent conjunctival haemorrhages should be instructed to avoid anticoagulants such as warfarin or non-steroidal antiinflammatory agents that have an effect on platelet functionality such as ibuprofen and asprin unless required for other medical conditions considering the risks and benefits of such therapy.

#### Retina

Retinal vascular malformations such as arterio-venous fistula, angiectasia, phlebectasia, and angioma have been described with a variable percentage from 0 to 10% [14, 16, 53]. However, retinal telangiectasia is reported with different frequencies based on the modality of examination of patients. Geisthoff et al. reported retinal telangiectasia in 1 of 75 patients examined [15]. These authors concluded that the prevalence of retinal telangiectases (< 1.3%) did not justify ophthalmological screening procedures. Similarly, Gòmez-Acebo et al. found a 1.12% prevalence of retinal involvement but acknowledged that subtle vascular retinal abnormalities may have been overlooked as patients only underwent ophthalmoscopic examination [49]. Retinal imaging has given new insight on retinal telangiectasia. Fluorescein angiography (FFA) is a method used for the evaluation of retinal circulation by administering an intravenous bolus of fluorescein sodium salt and taking serial photographs of the vascular network of the retina. Brant et al. carried out FFA in 2 of 20 patients (10%) with retinal telangiectasia; one patient had telangiectasia adjacent to the optic disc in one eye and in the papillomacular bundle in the fellow eye without leakage in any frame of angiography. A second patient showed fine telangiectasia throughout the retina [14]. Mennel et al. also performed FFA and described parafoveal telangiectasia in both eyes of one patient and a choroidal neovascular membrane with leakage in another patient. This patient was successfully treated in 2005 with photodynamic therapy (prior to the widespread first choice use of anti-VEGF therapy in ophthalmology for neovascular membranes); leakage and exudation resolved and visual acuity remained stable with follow up at one year. The authors, however, acknowledged that the idiopathic parafoveal telangiectasia could have been a coincidental finding or in the spectrum of HHT and speculated the possible influence of endoglin in the pathogenesis [54]. The incidence of retinal alterations has changed through recent studies by Sindhar et al. who examined eighteen patients with HHT through wide-field FFA. The authors found 83% of patients with peripheral telangiectasis with capillary dilatation and tortuosity in the nasal and temporal retina but no vascular or structural alterations within the macula. They suggested that retinal vascular malformations might be easily overlooked with fundus examination or fundus photography [48].

As FFA involves the use of dye and is a relatively invasive method, this technique may not be feasible in all cases. Near infrared imaging uses a light source with a wavelength of approximately 850 nm and for screening purposes wide-field NIR imaging may be a more practical method of screening. NIR imaging is now widely accessible in ophthalmology and has been recently used to observe microvascular retinal vascular abnormalities, as a further biomarker, in neurofibromatosis type 1 that are difficult to detect on ophthalmoscopic examination [55, 56]. Although Rinaldi et al. used NIR imaging in HHT [8] they did not comment on any vascular alterations, probably because the instrument provides images limited to the posterior pole, whereas telangiectasia are commonly located in the peripheral retina. NIR imaging with a wide-field modality could be promising as a screening tool as it is a rapid and non-invasive imaging method available in all major hospital facilities.

Retinal pigmentary alterations overlying choroidal ectatic vessels were reported by Tsai et al. The authors suggested that the altered choroidal vessels cause alterations of the retinal pigment epithelium (RPE) possibly owing to micro-exudation from ectactic vessels [54, 57]. Rinaldi et al. used infrared and red free images and found RPE alterations in three of 8 patients. In particular, they found atrophy temporal to the macula between the superior and inferior vascular arcades in areas corresponding to retinal telangiectasia in one patient; atrophy of the RPE-coriocapillaris complex of the posterior pole excluding the macula in both eyes of a second patient; and subtle changes of the peripheral RPE and defined areas of choriocapillaris and RPE atrophy extending from the posterior pole to the mid-periphery in correspondence of the vascular bed in a third patient [8].

Spectral domain optical coherence tomography (SD-OCT) is a non-invasive, dye free technique that uses a light source with a wavelength of approximately 850 nm, with maximum resolution sensitivity to obtain visualisation of the retinal layers and the choroid. Rinaldi et al. reported SD-OCT features of RPE hypo-reflectivity, with visible choroidal vessels, large choroidal vessels, and choroid hyper-reflectivity features in 3 of 8 patients [8].

A further development in ophthalmological imaging is optical coherence tomography angiography (OCT-A). This is a non-invasive high resolution imaging technique that enables to visualise the retinal and coroideal circulation. This method enables quantitative and qualitative evaluation of the superficial and deep retinal vascular plexuses, the choriocapillaris vessel density, and the foveal avascolar zone. Sindhar et al. performed OCT-A analysis evaluating the mean foveal avascular zone and foveal/parafoveal vessel density, however, they did not find vascular or architectural changes in the macular area. This is probably because HHT manifests with peripheral telangiectasia, and the posterior pole is not involved [48].

Retinal telangiectasia in patients with HHT are stable, and seldom cause symptoms, therefore, prophylaxis or treatment is not required. However, these are additional signs that can help in the early detection of signs of HHT.

#### Choroid

As the choroideal circulation is difficult to observe when the pigmented epithelium is intact, indocyanine green angiography (ICG-A), carried out with a water-soluble dye with high protein binding capacity that does not pass through the fenestrations of the choriocapillaris, enables choroidal circulation visualisation owing to the ability of infrared radiation emitted by the indocyanine to penetrate through normal ocular pigments. Tsai et al. performed ICG-A in one patient and found dilatation and tortuosity of choroidal vessels [57]. They found ectatic choroidal vessels that showed intense hyperfluorescence with adjacent hypofluorescence in the early frames extending from the peripapillary zone to the peripheral retina. They did not report leakage of dye in any frame and suggested that the altered vessels have an intact barrier. Rinaldi et al. reported ICG-A features consisting of large choroidal vessels, diffuse moderate leakage, choriocapillaris atrophy in the mid-periphery, and isolated choroidal telangiectasia in 3 of eight patients [8].

The genes implicated in HHT are critical for blood vessel development and response to TGF-β signalling [3]. Owing to choriocapillaris atrophy findings together with RPE alterations in HHT patients, Rinaldi et al. speculated that RPE atrophy causes choriocapillaris loss and photoreceptor degeneration or alternatively choroidal vascular insufficiency results in dysfunction of the RPE and photoreceptor degeneration [8]. This is supported by the findings of Schlingemann who reported that TGF-β is also a neurotrophic factor involved in maintaining the physiological function of the RPE and its connections with the retinal cells and the choriocapillaris, giving trophic support and contributing to the angiogenic balance [58].

Treatment is not indicated for choroidal vascular anomalies; however, choroidal haemorrhages have been reported during phacoemusification for cataract surgery and during vitrectomy for retinal detachment [53, 59]. Thus, during ocular operations the risk of choroidal haemorrhage seems to be elevated, and recognition of this severe complication is fundamental in order to take appropriate precautions in planning surgery.

#### Rare ophthalmic manifestations

Rare reports are by Kuchtey et al., who described a case of open angle glaucoma in a patient and his sister with type II HHT [60]. The authors suggested that this finding could be related to the disease, as it is caused by mutations in genes involved in the TGF-β superfamily signalling, which also plays a role in glaucoma pathogenesis. Van Went et al. described a case of spontaneous thrombosis of an orbital AVM leading to proptosis, chemosis, and ocular hypertension as the presenting symptom in one patient [61]. In one case, longstanding hypoxemia and polycythemia due to a large pulmonary AVM with right-to-left shunt, contributed to hyperviscosity and consequent branch retinal artery occlusion in a patient with HHT [62].

## Conclusions

HHT is a rare disease with a low but existing rate of life-threatening complications. The disease is frequently under-diagnosed as it typically relies on clinical manifestations. Ophthalmic signs are not included in the diagnostic criteria but their detection can lead to early diagnosis of HHT. The ophthalmologist can aid in detecting telangiectasia of the conjunctiva or the retina owing to the wide availability of advanced imaging methods. NIR may be a promising imaging modality to detect peripheral telangiectasia as retinal or choroidal biomarkers of HHT. Molecular diagnostics can successively be used to confirm diagnosis enabling to inform families of the possibility of further screening and early treatment.

## Data Availability

Not applicable.
